# Differential expression between drought-tolerant and drought-sensitive sugarcane under mild and moderate water stress as revealed by a comparative analysis of leaf transcriptome

**DOI:** 10.7717/peerj.9608

**Published:** 2020-07-28

**Authors:** Wanapinun Nawae, Jeremy R. Shearman, Sithichoke Tangphatsornruang, Prapat Punpee, Thippawan Yoocha, Duangjai Sangsrakru, Chaiwat Naktang, Chutima Sonthirod, Warodom Wirojsirasak, Kittipat Ukoskit, Klanarong Sriroth, Peeraya Klomsa-ard, Wirulda Pootakham

**Affiliations:** 1 National Omics Center (NOC), National Science and Technology Development Agency (NSTDA), Thailand Science Park, Pathum Thani, Thailand; 2 Mitr Phol Sugarcane Research Center Co., Ltd., Phu Khiao, Chaiyaphum, Thailand; 3 Department of Biotechnology, Faculty of Science and Technology, Thammasat University (Rangsit Campus), Pathum Thani, Thailand

**Keywords:** Sugarcane, Drought, Drought tolerance, Early response, Transcriptome, RNA-Seq

## Abstract

Sugarcane contributes 80% of global sugar production and to bioethanol generation for the bioenergy industry. Its productivity is threatened by drought that can cause up to 60% yield loss. This study used RNA-Seq to gain a better understanding of the underlying mechanism by which drought-tolerant sugarcane copes with water stress. We compared gene expression in KPS01-12 (drought-tolerant genotype) and UT12 (drought-sensitive genotype) that have significantly different yield loss rates under drought conditions. We treated KPS01-12 and UT12 with mild and moderate water stress and found differentially expressed genes in various biological processes. KPS01-12 had higher expression of genes that were involved in water retention, antioxidant secondary metabolite biosynthesis, and oxidative and osmotic stress response than UT12. In contrast, the sensitive genotype had more down-regulated genes that were involved in photosynthesis, carbon fixation and Calvin cycle than the tolerant genotype. Our obtained expression profiles suggest that the tolerant sugarcane has a more effective genetic response than the sensitive genotype at the initiation of drought stress. The knowledge gained from this study may be applied in breeding programs to improve sugarcane production in drought conditions.

## Introduction

Sugarcane (*Saccharum* ssp.) is a C4 grass in the Poaceae family. Modern sugarcane cultivars are mainly hybrids of sugar rich *Saccharum officinarum* species and the wild species *S. spontaneum* (Shearman et al., 2016). This plant is one of the most important economic crop species of the world as it is used in both the food and energy industries (Ferreira et al., 2017). The global harvest area and production of sugarcane have continually increased since 1961 and reached a maximum in 2013 (http://www.fao.org). The major planting areas of sugarcane are distributed in tropical and subtropical regions because the plant requires hot weather (32–38 °C) and relatively high rainfall (1,000–1,500 mm/year). Dry conditions can have positive effects on sucrose yield of sugarcane during maturation phase (Ferreira et al., 2017). However, stem and leaf growth of sugarcane is very sensitive to water stress during germination, tillering and during the vegetative growth phase (Ferreira et al., 2017). Drought can cause up to 60% yield loss in rainfed areas (Ferreira et al., 2017).

Selection and breeding of sugarcane cultivars that can adapt to drought conditions offers a promising solution to the problem. However, this is not trivial because the responses of sugarcane to drought can cause both positive and negative effects (Ferreira et al., 2017). For example, although drought-induced stomatal closure can help a plant prevent water loss, it can also cause photosynthetic inhibition (Wang et al., 2016). In addition, different sugarcane cultivars likely have different drought response mechanisms. Even if the mechanisms are complementary, the efficiency levels can vary among genotypes leading to poor combination results (Fracasso, Trindade & Amaducci, 2016). Therefore, it is imperative to have comprehensive data to understand the complex drought responses of sugarcane. Several molecular techniques have been applied to discover genetic responses of sugarcane to drought. Suppression subtractive hybridization identified differentially expressed genes (DEGs) in sugarcane cultivar Co740 under water deficit conditions by comparing to a condition with adequate water supply over a 45-day time course (Prabu et al., 2011). The DEGs appeared to be involved in many cellular processes including photosynthesis, signaling, molecular metabolisms and stress responses. Another gene expression study using microarrays revealed that the drought-tolerant SP83-5073 had fewer number of DEGs than the drought-sensitive SP90-1638 under mild (~80% substrate water content), moderate (40%) and severe (20%) water stress conditions (Rodrigues, De Laia & Zingaretti, 2009). More recently, a study applied RNA sequencing (RNA-seq) to de novo assemble the transcriptome and compare the level of gene expression between drought-tolerant (GN18) and drought-sensitive (FN95-1702) sugarcane cultivars exposed to drought stress (Xu et al., 2018). GN18, which was an offspring of FN95-1702, had higher expression levels of genes involved in photosynthesis, signal transduction and reactive oxygen species (ROS) scavenging.

RNA-Seq is a technique that uses high-throughput sequencing of transcript reads to identify gene expression changes. However, the high polyploidy complexity of the sugarcane genome can interfere with read mapping that is required to get gene expression from RNA-Seq. A transcriptome reference was often used for mapping sugarcane reads even though the mapping rate was moderate (Kasirajan et al., 2018). The recently reported monoploid reference genome of sugarcane (Garsmeur et al., 2018) enables a reference-based analysis of RNA-Seq data to yield precise gene expression profiles. Moreover, several sophisticated RNA-Seq data analysis softwares, for example, DEseq2 (Love, Huber & Anders, 2014), have been developed. In this study, we conducted RNA sequencing and analyzed the data by using the monoploid reference genome (Garsmeur et al., 2018) and DEseq2 to find gene expression change in a drought-tolerant sugarcane (KPS01-12) in response to the water stress. We compare the expression changes with those in a drought-sensitive sugarcane (UT12) to show genes that are uniquely and/or highly expressed only in the drought-tolerant sugarcane.

## Materials and Methods

### Plant material and growth conditions

To investigate the effects of drought stress on gene expression, the experiment was conducted in a naturally lit glasshouse at the National Science and Technology Development Agency, Pathum Thani, Thailand from May to July 2018. Sugarcane (*Saccharum* spp. hybrid KPS01-12 and UT12) plantlets were produced in tissue culture at Mitr Phol Sugarcane Research Center Co., Ltd., Chaiyaphum, Thailand and transplanted into potting trays (5 × 6 wells) on May 2018. The permission and regulation to obtain the sugarcane plant material were not required and no protected species was involved in this study. Thirty-day-old plants with ~10–12 cm stem height were transplanted into 1.5 L pots with peat and sand (50:50 v/v) for 28 days before applying the stress treatment. Each pot consisted of approximately 2,000 ± 50 g of peat and sand (50:50 v/v) and contained one plant. A nutrient solution was applied every fortnight through irrigation. The transplanted seedlings were grown at 35 ± 2 °C with 50% ± 10% humidity for 4 weeks and uniform seedlings were chosen for the study.

### Water stress treatment

In this experiment, the well-watered (WW) plants continued to receive full irrigation where field capacity (FC) was kept at 100%. For treatment, water stress (WS) was imposed by gradually withdrawing irrigation over a period of 14 days until the potting mix reached 50% FC, determined gravimetrically (Earl, 2003). Leaf samples were collected from the well-watered and water-stressed plants at seven and fourteen days after the onset of the treatment corresponding to ~75% FC (mild WS) and ~50% FC (moderate WS), respectively.

### Field conditions for yield data collection

We collected yield data from Mitr Phol Sugarcane Research Center Co., Ltd. at Phukhieo District, Chaiyaphum Province during December 2017–December 2018, which is one of the growing seasons of the area. Sugarcane plants were grown under normal water condition and dry condition. The plants under normal water condition were supplied with water of ~60 cubic/rai twice a month from December 2017 to May 2018 while the plants under dry condition did not receive any water.

### RNA extraction, cDNA library construction and sequencing

Two biological replicates were used for each genotype, water treatment, and time point. All samples were pulverized in liquid nitrogen, and then put into CTAB extraction buffer (2% CTAB, 1.4 M NaCl, 2% PVP, 20 mM EDTA pH 8.0, 100 mM Tris-HCl pH 8.0, 0.4% SDS). Total RNA was extracted from the aqueous phase twice using 24:1 chloroform:isoamylalcohol, and the supernatant was precipitated overnight in 1/3 volume of 8 M LiCl. RNA pellets were washed with 70% ethanol, air-dried, and resuspended in RNase-free water. Dynabeads mRNA purification kit (Thermo Fisher Scientific, Waltham, MA, USA) was used for mRNA enrichment. RNA integrity was assessed using Fragment Analyzer (Advanced Analytical Technologies, Inc., Ames, IA, USA). cDNA libraries were prepared according to the Ion Total RNA-Seq Kit v2 (Life Technologies, Carlsbad, CA, USA) protocol. The template-positive Ion 540^™^ ion sphere particles (ISPs), containing amplified cDNA (about 200 bp), were prepared using the Ion 540^™^ kit-OT2 kit and the reaction was performed in the OneTouch^™^ 2 Instrument. The template-positive ISPs were enriched using Ion OneTouch^™^ ES. The libraries were sequenced using the Ion 540^™^ chip on the Ion S5 XL sequencing system (Thermo Fisher Scientific, Waltham, MA, USA).

### Identification of differential expression gene and functional clustering

Raw reads were processed by trimming sequencing adapters and low-quality bases. Possible ribosomal RNA contamination in the data set was removed by comparing to SILVA rRNA database (release 132) using local BLAST search (Camacho et al., 2009; Quast et al., 2012).

Cleaned reads were mapped to the sugarcane monoploid reference genome assembly using STAR aligner (version 2.6.1a) (Dobin et al., 2013). The genome sequence, protein sequences and GFF annotation files were downloaded from http://sugarcane-genome.cirad.fr (Garsmeur et al., 2018). Raw counts of the reads mapped to gene regions were quantified by HT-seq count (version 0.9.1) (Anders, Pyl & Huber, 2015). The reads mapped to multiple loci were discarded. Differentially expressed genes (DEGs) were identified using R package DESeq2 (Love, Huber & Anders, 2014) with Benjamini-Hochberg false discovery rate adjusted *p*-value of 0.05. For samples collected under mild or moderate WS, two sets of DEGs were calculated from two comparison types. The first set compared water-stressed and well-watered samples of the same genotype (WS/WW comparison). The second set compared two genotypes (KPS01-12/UT12 comparison) and DEGs that are uniquely present under WS condition were selected. For both comparison types, only identified DEGs with log2 fold-change greater than 1.5 were retained for subsequent analyses. Two biological replicates were enough for DEseq2 normalization and statistic test to keep sensitivity in detecting differential gene expression when a foldchange cutoff of 1.5 is used (Schurch et al., 2016).

Gene symbols and protein products for the DEGs were obtained from GFF annotation file provided along with the reference genome sequence. The DEGs were clustered based on their possible biological functions or pathways in which they were involved by performing similarity search of corresponding protein sequences with Mercator v.3.6 (Lohse et al., 2014), AgriGO v2.0 (Tian et al., 2017), KEGG database (Kanehisa & Goto, 2000) and UniProt database (UniProt, 2008) depending on the data available for each DEG.

### Quantification of target gene transcripts with NanoString nCounter SPRINT Profiler

A custom-designed NanoString nCounter gene expression codeset targeting 15 sugarcane genes and five housekeeping genes was applied to validate the RNA-seq results. The target genes were selected from the DEGs obtained from the RNA-seq data and the housekeeping genes were selected based on (De Andrade et al., 2017). For each sample, 100 ng of total RNA in 5–8 µl solution was used in the hybridization reaction according to the manufacturer’s protocol. Purified target RNA-probeset complexes were loaded onto nCounter Cartridges and were quantified on nCounter Sprint Profiler. We used nSolver 4.0 software (https://www.nanostring.com/products/analysis-software/nsolver) to analyse the data, in which eight negative and five positive control genes provided by the manufacturer were used for background noise removal and technical variability normalization, respectively.

## Results

### Phenotypic responses to water stress

The field data collections revealed that KPS01-12 (25% yield reduction) had significantly less yield loss than UT12 (66% yield reduction) under drought conditions while their yields under normal water conditions were similar (Table S1). The data revealed that KPS01-12 was more tolerant to drought than UT12.

### Gene expression changes in response to water stress

RNA sequencing using Ion S5 XL system yielded a total of 12 Gb of transcriptomic sequences from two sugarcane genotypes with two biological replicates for each of mild WS, moderate WS and WW conditions (Table S2). We mapped our RNA reads to the genome of *Sorghum bicolor*, *S. spontaneum* and *Saccharum* spp. hybrid cultivar R570 (Garsmeur et al., 2018). The average percentage over all samples of RNA reads that could be mapped to a unique locus on the R570 sugarcane genome was 67% while the percentage of RNA reads that were mapped to multiple loci was 3% and the percentage of the unmapped reads was 26% (Table S3). The average percentage of the uniquely mapped, multiple loci mapped and unmapped reads with the *S. bicolor* genome were 50%, 22% and 11%, respectively. With the *S. spontaneum* AP85-441 genome, the average percentages were 34%, 30% and 31%, respectively. The mapping results with the R570 sugarcane genome were used in our analysis of gene expression since it had the highest percentage of uniquely mapped reads. The average number of genes that had at least one uniquely mapped read was approximately 15,000 genes, accounting for 61% of all annotated protein coding genes in the reference genome. The sequencing data have been deposited in the NCBI Sequence Read Archive database under the accession number PRJNA574856.

**Figure 1:**
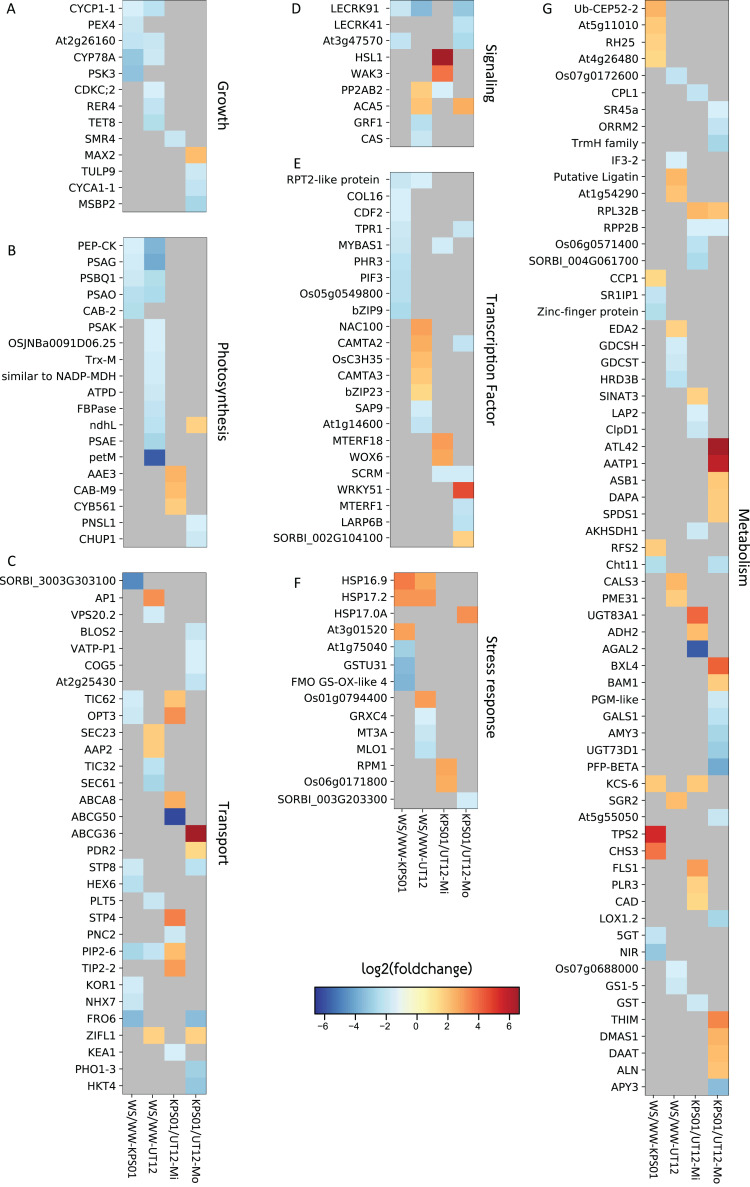
Differentially expressed genes in sugarcanes under the water-stressed treatments. The heat maps show log2 of the fold change of non-redundant DEGs that are classified into seven classes of (A) growth, (B) photosynthesis, (C) molecular transport, (D) signaling, (E) transcription factor, (F) stress response, and (G) metabolism-related genes. Heat map columns that are labeled as WS/WW-KPS01 (lane 1) and WS/WW-UT12 (lane 2) show log2 of the fold change of DEGs obtained from WS/WW comparisons for KPS01-12 and UT12, respectively. Heat map columns that are labeled as KPS01/UT12-Mi (lane 3) and KPS01/UT12-Mo (lane 4) show log2 of the fold change of DEGs obtained from KPS01-12/UT12 comparisons at mild WS and moderate WS, respectively. Colors scaled from blue to red show log2 of fold change levels from up-regulation to down-regulation. Cells with dark grey color show insignificant expression changes of a gene at a specific condition. The full name of each DEG is shown in Table S4.

**Figure 2:**
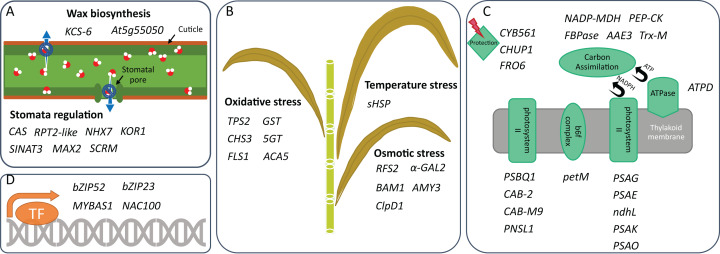
Drought tolerance activities in which DEGs are involved. Genes that can participate in (A) drought-related stress response, (B) water retention, (C) photosynthesis, and (D) transcription regulation of two sugarcane genotypes are shown. These genes are identified under mild and/or moderate water stresses and are selected for discussion based on their activities reported in other plants.

Multiple genes were significantly differentially expressed in response to mild or moderate water stress (Figs. 1 and 2; Table S4). Six sets of DEGs were calculated from mild WS and moderate WS compared to the WW condition for each genotype and by comparing KPS01-12 to UT12 for each of mild WS and moderate WS. We filtered out DEGs that had been annotated to encode hypothetical or redundant proteins in the reference genome and focused only on genes with known functions. From the WS/WW comparison, KPS01-12 under mild WS and moderate WS had four and 44 final DEGs, respectively, and UT12 had two and 58 DEGs, respectively (Fig. 1; Table S4). From the KPS01-12/UT12 comparison, the final number of DEGs under mild and moderate WS were 39 and 56, respectively. Of these DEGs, 58% (*n* = 23) and 37% (*n* = 21) had higher expression in the tolerant genotype than the sensitive genotype under mild WS and moderate WS, respectively.

To validate the RNA-Seq results, we used NanoString to directly quantify the mRNA of 15 genes from the DEG list. We computed log2 fold-change in the same six comparison groups as in the RNA-seq experiment. Ten of these genes were up-regulated with the log2 fold-change values between 1.45 and 6.87 while the other five genes were down-regulated with the log2 fold-change from −2.30 to −3.13. The log2 fold-change values were consistent with the results obtained from the RNA-seq data (Fig. S1).

All non-redundant DEGs were classified into seven major classes of biological processes (Fig. 1; Table S4). Growth and photosynthesis were the only classes where all DEGs from WS/WW comparison of both genotypes were down-regulated. For the molecular transport class, the DEGs were involved in vesicle, lipid, protein, sugar, ion, and metabolite transport (Fig. 1C; Table S4). For genes in signaling and transcription factor (TF) classes, the tolerant genotypes had only down-regulated DEGs from the WS/WW comparison (Figs. 1D and 1E). In the stress-responsive class, most DEGs were involved in heat and oxidative stress responses (Fig. 1F; Table S4). The cellular metabolism class contained the highest number of DEGs among all DEG classes (Fig. 1G), but none of these DEGs was shared between the two genotypes. The cellular metabolism DEGs were involved in RNA binding and processing, and metabolism of protein, carbohydrate, lipid, secondary metabolite and organonitrogen compounds (Table S4).

## Discussion

### RNA mapping rates with different reference genomes

Mapping RNA reads to the R570 sugarcane genome yielded the highest percentage of uniquely mapped reads compared to the *S. bicolor* or *S. spontaneum* genomes. Sugarcane cultivar R570 has whole *S. officinarum* chromosomes, whole *S. spontaneum* chromosomes and *S. officinarum*/*S. spontaneum* recombinant chromosomes (Garsmeur et al., 2018; D’Hont et al., 1996). The RNA reads of KPS01-12 and UT12, which are hybrid cultivars, may be more compatible with the R570 sugarcane genome than the other two reference genomes. Additionally, the reference sequence of the R570 sugarcane genome was released as a monoploid genome, which can prevent RNA reads from being mapped to multiple loci.

### Higher expression of stomata-regulating and wax-biosynthetic genes in the drought-tolerant genotype than the drought-sensitive genotype

Avoidance is a strategy to reduce the amount of water loss by, for example, limiting stomata opening and/or biosynthesizing wax to cover organ surface (Ferreira et al., 2017). Under mild and moderate water treatments, we identified different expression profiles of several genes that were reported in other plants to regulate stomata opening or closure. KPS01-12 down-regulated expression of *RPT2-like* gene that was reported to regulate stomatal opening (Inada et al., 2004). UT12 also had down-regulation of this gene, but not as low as KPS01-12. Moreover, KPS01-12 had higher expressions of *SINAT3* and *MAX2*, which may be involved in stomatal closure (Bao et al., 2014; Bu et al., 2014), than the sensitive genotype (UT12). In *Arabidopsis, SINAT* could increase stomatal closure under drought stress and ABA treatments (Bao et al., 2014) while defective *MAX2* caused larger stomatal aperture, faster water loss and reduced cuticle thickness (Bu et al., 2014). *Arabidopsis* with *MAX2* mutations were more sensitive to osmotic and drought stresses than wild type (Bu et al., 2014). Therefore, the up-regulation of these genes likely contributes to the positive drought response in KPS01-12.

In addition to stomata regulation, KPS01-12 had up-regulation of *KCS-6* expression, which was involved in wax and suberin biosynthesis and could prevent water and yield losses (Xue et al., 2017). Expression level of *KCS-6* in KPS01-12 was 3.6 folds higher than UT12. The up-regulation was observed when KPS01-12 was under mild WS, suggesting that it may be an early avoidance mechanism that helps the tolerant genotype respond to mild water stress. The sensitive genotype had higher expression of *At5g55050* encoding GDSL-lipase-like protein than the tolerant genotype. In barley, a GDSL motif lipase played roles in leaf water retention associated with deposition of cutin polymer in the leaves (Li et al., 2017). The results suggest that both genotypes likely have different non-stomatal mechanisms. However, the transcriptional response is faster in the tolerant genotype than the sensitive genotype.

### Unique expression of genes in secondary metabolite biosynthesis pathways and high expression of oxidative and osmotic stress responsive genes of the drought-tolerant genotypes

The tolerance strategy is the ability to alleviate the effects of drought-associated stresses (Ferreira et al., 2017). Drought causes oxidative, osmotic and temperature stress (Salehi-Lisar & Bakhshayeshan-Agdam, 2016). KPS01-12 had high expression level of genes involved in the biosynthesis of terpenoids (41-fold up-regulated putative *TPS2*) and flavonoids (14-fold up-regulated *CHS3* and *FLS1*) (Fig. 1; Table S4). Function of these secondary metabolites as antioxidant has been reported. For example, terpenoids prevent photosynthetic apparatus from drought-related oxidative stress (Kleiber et al., 2017; Haberstroh et al., 2018). Flavonoids are direct scavengers of hydrogen peroxide, singlet oxygen, and hydroxyl radicals which are harmful to biomolecules (Das & Roychoudhury, 2014). Flavonoids have also been reported as secondary ROS scavengers in photosynthetic apparatus that receive excess excitation energy (Fini et al., 2011). It was additionally suggested that H_2_O_2_ in the chloroplast under severe excess light conditions is transported by tonoplast intrinsic proteins (TIP) to the vacuole which is the storing site for flavonoids (Fini et al., 2011). In this study, the tolerant genotype had higher expression of aquaporin *TIP2* than the sensitive genotype.

The tolerant genotype had a 6.2-fold higher expression of *ACA5* than the sensitive genotype. In transgenic tobacco, overexpression of *OsACA5* (or *OsACA6* as alternative name) reduced H_2_O_2_ accumulation and water loss while improve photosynthetic efficiency, root and shoot growth, survival rate and proline accumulation when compared to the wild-type (Huda et al., 2013). In addition, the tolerant genotype had down-regulated expression of *5GT* encoding glucosyltransferase that added UDP-glucose to the 5′-hydroxyl group of anthocyanin, by which the ROS scavenging ability of anthocyanin is decreased (Zhao et al., 2014). It also had down-regulated *GST* encoding glutathione S-transferase that reduced glutathione (GSH), which is required for ROS elimination (Das & Roychoudhury, 2014), to detoxify xenobiotic substances. The *A. thaliana AtGSTU17*-knockout plants showed higher GSH level, better redox potential, and were more tolerant to drought and salt stresses than wild-types (Chen et al., 2012). Down-regulation of *5GT* and *GST* may help KPS01-12 to preserve antioxidant molecules in the form that can neutralize ROS. Expression profiles of all these genes can be beneficial to the drought-tolerant genotype to effectively reduce the effects of oxidative stress under drought. However, it is also possible that the tolerant genotype generates more ROS than the sensitive genotype under water stress, so that it requires more expression regulations of ROS-responsive genes.

For osmotic stress response, KPS01-12 had up-regulated expression of *RFS2* that participated in the production of raffinose, which was known as an osmoprotectant and reactive oxygen species (ROS) scavenger (Taji et al., 2002; Nishizawa, Yabuta & Shigeoka, 2008). In contrast, UT12 had a 52-fold higher expression of *α-GAL2* encoding alpha-galactosidase, which hydrolyzes raffinose and may reduce concentration of this osmoprotectant (Sengupta et al., 2015), than KPS01-12. For other sugar metabolizing genes, KPS01-12 had a 3.4-fold higher expression level of *BAM1* than UT12 but had a 6.8-fold lower expression of *AMY3*. In *A. thaliana* leaves, both *BAM1* (beta-amylase) and *AMY3* (alpha-amylase) were reported to play positive roles against osmotic stress by releasing sugar such as maltose and sugar-derived osmolytes in an ABA-dependent manner (Thalmann et al., 2016). The difference between these two genes was that a defective mutation of *BAM1* caused significantly reduced maltose concentration compared to control while *AMY3* mutation showed a delay in that reduction (Thalmann et al., 2016). Maltose was suggested to sustain the biosynthesis of proline (and soluble sugars) and, thereby, reduce osmotic and oxidative damage under drought stress (Zanella et al., 2016). The differences in *BAM1* and *AMY3* expressions may result in different maltose concentrations and osmotic stress response capacities between the tolerant and the sensitive genotype. Overall, the results show that the tolerant genotype has a higher expression of oxidative and osmotic stress responsive genes than the sensitive genotype.

### Higher expression of genes in carbon assimilation cycle of the tolerant genotype than the sensitive genotype and their different expressions of photodamage related genes under moderate water stress

Deficiency of water can negatively affect photosynthesis and cause tremendous yield loss (Gururani, Venkatesh & Tran, 2015; Fahad et al., 2017). In this study, we found that the number of down-regulated photosynthetic genes in UT12 was higher than KPS01-12 (Fig. 1; Table S4). Four genes were found to be down-regulated in both genotypes, but the fold change was higher in UT12 than KPS01-12. One of these genes was *pckA* where KPS01-12 had 2.9-fold down-regulation while UT12 had 12.2-fold down-regulation (Fig. 1; Table S4). There is a report shows that *pckA* encodes phosphoenolpyruvate carboxykinase (PEP-CK), which plays roles in the decarboxylation pathway. The sugarcane plant, especially drought-tolerant genotypes, uses decarboxylation pathway as an alternative to the NADP-ME pathway for carbon assimilation under water deficit conditions (Fahad et al., 2017; Cacefo et al., 2019). KPS01-12 also had higher expression of *AAE3* than UT12. Plants use oxalyl-CoA synthetase encoded by *AAE3* to convert oxalate to CO_2_ (Foster et al., 2012). *Amaranthus hybridus* (C4 weed) uses CO_2_ from calcium oxalate degradation as a subsidizing carbon source under drought and CO_2_ starvation conditions (Tooulakou et al., 2016). Additionally, the sensitive genotype down-regulated a gene similar to *NADP-MDH* and *FBPase*, which are required for the Calvin cycle. These results imply that expression of genes in carbon assimilation process of UT12 is more sensitive to the water stress than KPS01-12, which may explain the greater yield loss under drought of the sensitive genotype (Table S1).

Under drought, plants continuously absorb light energy while the availability of water is limited. Excess light can unbalance electron excitation and utilization and results in an increased accumulation of ROS (Das & Roychoudhury, 2014). Plants have an array of mechanisms to alleviate the effects of excess light energy. Our results showed that KPS01-12 had higher expression of *CYB561* than UT12. This gene encodes transmembrane ascorbate required for safely dispersing excessive energy absorbed by chloroplasts under severe drought of wild watermelon (Nanasato, Akashi & Yokota, 2005). On the other hand, UT12 had a higher level of *CHUP1* than KPS01-12. This gene participates in chloroplast movement to avoid ROS generation and photodamage under imbalanced light/photosynthetic conditions (Kasahara et al., 2002). These results show that the tolerant genotype seems to use a protection strategy (together with high expression of putative *TPS2* and *CHS3* genes) to prevent photosynthetic apparatus damage. In contrast, the sensitive genotype probably uses the avoidance strategy to hide chloroplasts from light.

### Down-regulation of particular transcription factor genes can be beneficial to the tolerant genotype in response to drought

The stress perceived by plants trigger cascades of signals and results in the regulation of gene expression via the control of transcription factors. From WS/WW comparison, KPS01-12 had only down-regulated transcription factor genes. Some of these genes, for example, *bZIP* (similar to *OsbZIP52/RISBZ5*) and *MYBAS1* (possibly R2R3 family) were reported to play either positive or negative roles in plants under drought (Baldoni, Genga & Cominelli, 2015; Liu, Wu & Wang, 2012). Rice overexpressing *OsbZIP52* showed down-regulation of stress-related genes and significantly increased sensitivity to drought (Liu, Wu & Wang, 2012). Sugarcane R2R3-MYB was reported to be involved in an ABA-mediated leaf senescence signaling pathway (Guo et al., 2017) and expression of one of its isoforms could reduce biomass accumulation (Fávero Peixoto-Junior et al., 2018). In contrast, *NAC100*, which was up-regulated in UT12, was reported to cause early-senescence and positively regulated chlorophyll-degrading genes (Oda-Yamamizo et al., 2016). Considering the higher drought durability of KPS01-12 than UT12, the expression profile of these TFs is likely a part of the positive response of the tolerant genotype to the water stress.

## Conclusions

This study compared gene expressions between a drought tolerant sugarcane KPS01-12 genotype and a drought sensitive sugarcane UT12 genotype. Our experiment was conducted under mild and moderate water stresses to identify primary transcriptional response of the tolerant sugarcane to drought. From RNA-Seq data, we could identify gene expression changes between KPS01-12 and UT12 from a variety of pathways. We found that KPS01-12 had different expression profiles of genes that were involved in water retention, secondary metabolite biosynthesis, oxidative and osmotic stress protection, photosynthesis, and transcription regulation from UT12. These positive gene expressions may contribute to the higher yield maintaining ability of the tolerant sugarcane genotype under drought than the sensitive genotype. The obtained information not only expand our understanding of the underlying mechanisms that a drought-tolerant sugarcane uses to cope with water stress but may also be applied in breeding programs that aim to improve sugarcane production in drought conditions.

## Supplemental Information

10.7717/peerj.9608/supp-1 Supplemental Information 1 Comparison of the gene expression foldchanges calculated with DESeq2 (RNA-seq data) and nSolver (nanoString data) software. The foldchanges for CH3 and HSP16.9_KPS01 were obtained from the WS/WW comparison of Kps01-12 samples under mild WS. The foldchanges for HSP16.9_UT12 was from the WS/WW comparison of UT12 samples under mild WS. The foldchanges for PSAG, bZIP52, At3g01520, GSTU31, P450, and PIP2-6_KPS01 were obtained from the WS/WW comparison of KPS01-12 samples under moderate WS. The foldchanges for NRX2 and PIP2-6_UT2 were from the WS/WW comparison of UT12 samples under moderate WS. The foldchanges for FLS1, OTP3, WOX6, AAE3 were from the KPS01-12 /UT12 comparison under mild WS and those for BXL4 and ABCG36 were from the KPS01-12 /UT12 comparison under moderate WS. The foldchanges were selected based on the Benjamini-Hochberg false discovery rate adjusted p-value of 0.05.

10.7717/peerj.9608/supp-2 Supplemental Information 2 Yield and related parameters measured under field condition for the drought-tolerant genotype (KPS01-12) and the drought-sensitive genotype (UT12).

10.7717/peerj.9608/supp-3 Supplemental Information 3 Details of RNA-seq data from Ion Proton and percentage of sequence matching with monoploid reference genome of sugarcane.

10.7717/peerj.9608/supp-4 Supplemental Information 4 Statistic of mapping RNA reads to three reference genomes.

10.7717/peerj.9608/supp-5 Supplemental Information 5 Significantly differentially expressed genes that were identified under mild and moderate water stresses.

## Additional Information and Declarations

### Data Availability

The following information was supplied regarding data availability: Data are available at NCBI Sequence Read Archive (SRA) database: PRJNA574856.
