# Supplementary figures and images for: Differential expression between drought-tolerant and drought-sensitive sugarcane under mild and moderate water stress as revealed by a comparative analysis of leaf transcriptome
Source: PeerJ. 2020 Jul 28;8:e9608. doi: 10.7717/peerj.9608 (PMC7676377; doi:10.7717/peerj.9608)

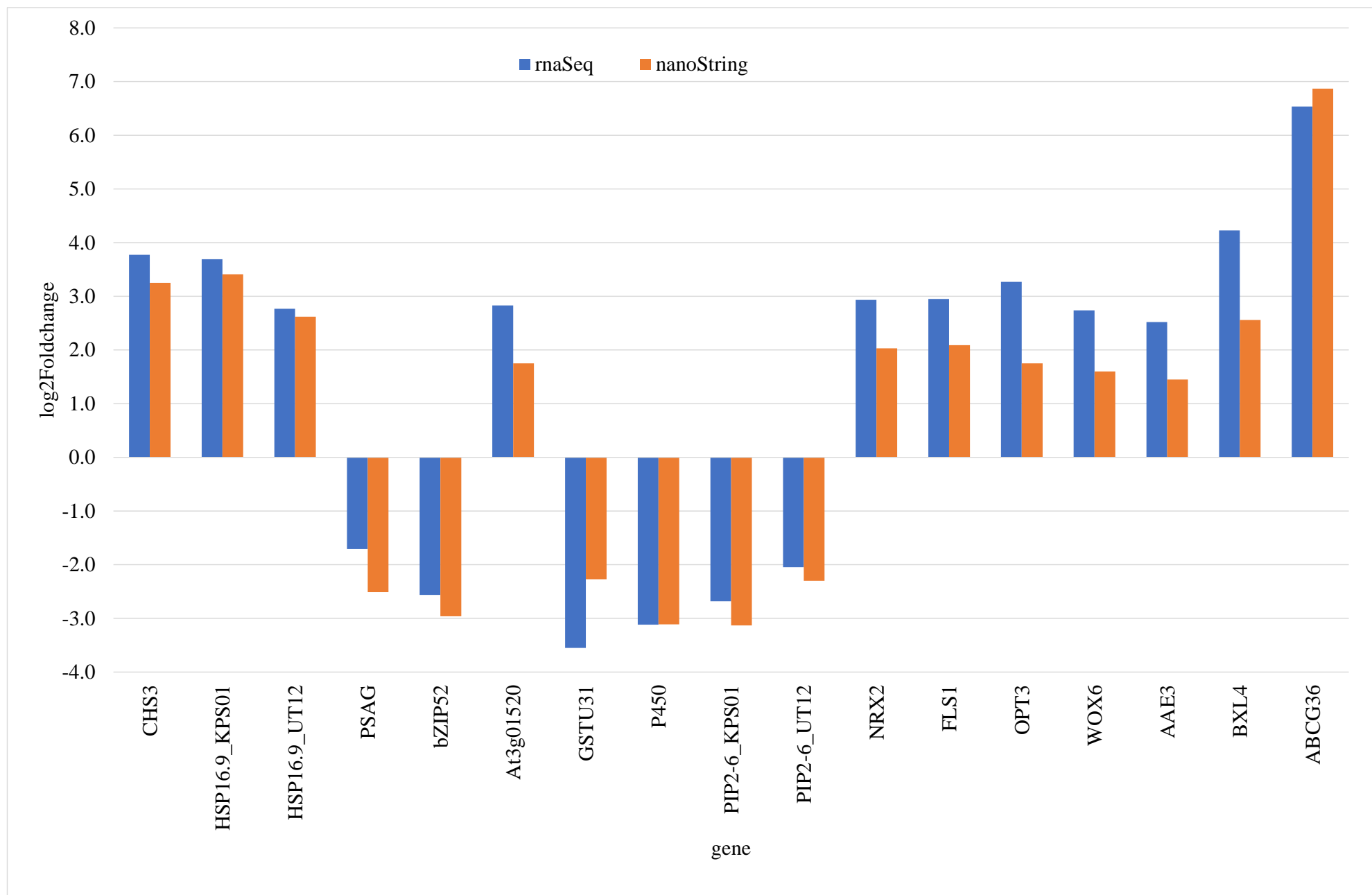

Supplement: Supplemental Information 1 — The foldchanges for CH3 and HSP16.9_KPS01 were obtained from the WS/WW comparison of Kps01-12 samples under mild WS. The foldchanges for HSP16.9_UT12 was from the WS/WW comparison of UT12 samples under mild WS. The foldchanges for PSAG, bZIP52, At3g01520, GSTU31, P450, and PIP2-6_KPS01 were obtained from the WS/WW comparison of KPS01-12 samples under moderate WS. The foldchanges for NRX2 and PIP2-6_UT2 were from the WS/WW comparison of UT12 samples under moderate WS. The foldchanges for FLS1, OTP3, WOX6, AAE3 were from the KPS01-12 /UT12 comparison under mild WS and those for BXL4 and ABCG36 were from the KPS01-12 /UT12 comparison under moderate WS. The foldchanges were selected based on the Benjamini-Hochberg false discovery rate adjusted p-value of 0.05. [file peerj-08-9608-s001.pdf]
